# Changing outpatient referral patterns in a small pediatric nephrology practice

**DOI:** 10.1186/s12887-018-1164-1

**Published:** 2018-06-19

**Authors:** Coral Hanevold, Susan Halbach, Jin Mou, Karyn Yonekawa

**Affiliations:** 1 Department of Pediatrics, Division of Nephrology, University of Washington, Seattle, WA USA; 2 MultiCare Institute for Research & Innovation, MultiCare Health System, Tacoma, WA USA

**Keywords:** Nephrology, Ambulatory referrals, Pediatrics

## Abstract

**Background:**

We have noted a large number of referrals for abnormal kidney imaging and laboratory tests and postulated that such referrals have increased significantly over time. Understanding changes in referral patterns is helpful in tailoring education and communication between specialists and primary providers.

**Methods:**

We performed a retrospective chart review of new patient referrals to Mary Bridge Children’s Nephrology clinic for early (2002 to 2004) and late (2011 to 2013) cohorts. The overall and individual frequencies of referrals for various indications were compared.

**Results:**

The overall number of new visits was similar for early (511) and late (509) cohorts. The frequency of referrals for solitary kidneys and multi-cystic dysplastic kidneys, microalbuminuria and abnormal laboratory results increased significantly (Odds Ratio (OR) and 95% Confidence Interval of OR: 1.920 [1.079, 3.390], 2.862 [1.023, 8.006], 2.006 [1.083, 3.716], respectively) over the time interval while the proportion of referrals for urinary tract infections (UTIs) and vesicoureteral reflux (VUR) decreased by half (OR: 0.472, 95% CI: 0.288, 0.633). Similarly, referrals for urinary tract dilation and hydronephrosis occurred significantly less often (8% versus 6%, OR: 0.737, 95% CI: 0.452, 1.204) with similar changes in referrals for voiding issues (OR: 0.281, 95% CI: 0.137, 0.575). However, these changes were not statistically significant. Frequencies for other indications showed little variation.

**Conclusions:**

Changes in indications for referral likely reflect evolution of practice in management of UTIs and VUR and increased use of imaging and laboratory testing by pediatric providers. These findings have relevance for ongoing education of pediatricians and support the need for collaboration between primary providers and nephrologists to assure the judicious use of resources.

## Background

For most pediatric nephrologists, outpatient care constitutes a significant volume of their clinical work. Primary care providers differ in regard to potential triggers for consultation with a pediatric nephrologist. This variation may be due to differences in training, experience, practice philosophy, patient volume, parental attitudes, among other factors. In turn, access to pediatric nephrologists is influenced by factors such as geography, financial considerations, sub-specialty practice approach, overlap with other specialists or other centers, center volume and availability of pediatric nephrologists [1–3]. Requirements for prior evaluation may vary from none to well-defined criteria, depending on the referral indication [4].

The increased availability and utilization of laboratory testing and imaging have been documented over the last two decades) [5–7]. Clinical guidelines for the evaluation and management of vesicoureteral reflux (VUR) have also changed during this time period [8, 9]. To evaluate how these changing practices may have impacted indications for referral to pediatric nephrology, we compared referral indications over two time periods, 2002–2004 and 2011–2013, to Mary Bridge Children’s, a general non-academic pediatric nephrology practice in the Pacific Northwest of the United States (US) with a catchment population of 549,000 children and adolescents under 18 years of age.

## Methods

This study was a retrospective chart review of children referred for outpatient evaluation at the pediatric nephrology practice at Mary Bridge Children’s during two 24-month time periods: early cohort (July 2002–June 2004) and late cohort (May 2011–April 2013). All visits were conducted by pediatric nephrologists (two and three for early and late cohorts, respectively) who varied between the two time periods. All new referrals for initial outpatient visits were included as well as a limited number of patients with known renal disease transferring care to our center from other programs. New inpatient consults and hospital follow-up visits were excluded. Patients were identified using the Current Procedural Terminology (CPT) codes: “new visits (99202- 99205) AND outpatient consultations (99242- 99245)” in the electronic medical record system. The primary care provider’s indication for referral was determined by reviewing documentation by the pediatric nephrologist and was categorized as shown in Table 1. If more than one problem was noted at the time of referral the primary indication leading to referral was used for classification. The indication for referral provided by referring provider differed at times from the diagnosis of the pediatric nephrologist, but for these analyses only referral indications were considered. Hematuria and proteinuria were considered together as the two conditions frequently occur concomitantly. Urinary tract infections (UTIs) and VUR were considered together and separately on subsequent analysis. Children referred for VUR could have a history of UTIs, but referral was prompted by the imaging finding.

**Table 1:** Indications for Referral

HBP	Elevated blood pressures/hypertension
HEM/PRO	Hematuria and/or proteinuria
UTI/VUR	Urinary tract infection and/or vesicoureteral reflux
ABN IMAGING	Includes solitary kidney, hydronephrosis, dilation of collecting systems, horseshoe kidney, duplicated collecting systems, isolated simple cysts, multicystic kidney, and other minor abnormalities such as abnormal size or appearance of one or both kidneys
↓GFR	Decreased glomerular filtration rate, acute or chronic, includes hemolytic uremic syndrome
STONES/NC	Stones/nephrocalcinosis and hypercalciuria
MICROALB	Microalbuminuria
GLOM/VAS	Glomerular disease or vasculitis, includes nephrotic syndrome, Henoch Schonlein purpura, hereditary nephritis, acute glomerulonephritis or vasculitis of any type
VOIDING ISSUES	Includes enuresis, urinary frequency or urgency, dysuria, polyuria, daytime incontinence
ABN LABS	Abnormal laboratory studies (urine or blood) excluding hypercalciuria, microalbuminuria
OTHER	Such as tuberous sclerosis, polycystic kidney disease, Bardel Biedel syndrome, Beckwith Wiedeman, prenatal counseling, edema, flank pain, family history of kidney disease

Pearson’s chi square tests were used to compare the overall composition and distribution of referrals between the two time periods as well as individual referral categories. Additional comparisons were performed considering referrals for abnormal imaging, using the following categories: solitary kidney/multicystic dysplastic kidney, urinary tract dilation or other abnormal finding (cysts, abnormal echogenicity or size, etc). These sub-analyses were also done using the Pearson’s chi square test. Taking the early cohort as the reference, risks for each specific referral reasons in the later cohort were calculated and odds ratio was presented with 95% confidence interval. Data were analyzed using Stata 14 (Stata Corp, College Station, TX). This study was fully approved by the MultiCare Health System Institutional Review Board (MHSIRB, IRB Study Number: 11.29). Due to its retrospective data review feature, no patient consent was requested.

## Results

The volume of new patients was similar in the two cohorts (511 in early, 509 in late cohorts respectively). Additionally, the distribution across age groups was consistent between the two time periods (data not shown). A summary of differences in referral indications between the time periods is displayed in Table 2. Overall the composition of the referral indications differed between the two time periods (*p* < .001). Looking at specific indications for referrals, abnormal laboratory results were a more frequent reason for referral in the late cohort as compared to the early cohort. Microalbuminuria was considered as a separate category and showed a significant increase from 1 to 3% though the absolute number of patients referred for this indication was small. Referral for voiding issues occurred much less frequently in the later cohort. Hematuria and/or proteinuria and elevated blood pressure or hypertension were the leading indications for referral in both cohorts and did not differ appreciably between the two time periods.

**Table 2:** Referral Indications for Early and Late Cohorts*

Indication for Referral	Early Cohort (2002–2004) *n* (%)	Late Cohorts (2011–2013) *n* (%)	*χ*^*2*^, *p*	Odds Ratio‡ [95% CI]
HBP	107 (20.9%)	120 (23.6%)	1.024, 0.311	1.165 [0.867, 1.565
HEM/PRO	108 (21.1%)	105 (20.6%)	0.040, 0.842	0.970 [0.717, 1.312]
UTIs/VUR*	87 (17.0%)	41 (8.1%)	18.698, 0.000	0.472 [0.288, 0.633]
ABN IMAGING	79 (15.5%)	96 (19.1%)	2.311, 0.128	1.287 [0.929, 1.784]
VOIDING ISSUES*	34 (6.7%)	10 (2.0%)	13.583, 0.000	0.281 [0.137, 0.575]
↓GFR	21 (4.1%)	18 (3.5%)	0.228, 0.633	0.855 [0.450, 1.625]
STONES/NC	17 (3.3%)	23 (4.5%)	0.961, 0.327	1.375 [0.726, 2.606]
GLOM/VAS	20 (3.9%)	30 (5.9%)	2.145, 0.143	1.538 [0.861, 2.745]
ABN LABS†	16 (3.1%)	31 (6.1%)	5.080, 0.024	2.006 [1.083, 3.716]
MICROALB†	5 (1.0%)	14 (2.8%)	4.380, 0.036	2.862 [1.023, 8.006]
OTHER	17 (3.3%)	21 (4.1%)	0.454, 0.501	1.250 [0.652, 2.399]

Overall frequencies within categories differed (*p* < 0.001 comparing cohorts)

*HBP* elevated blood pressure or hypertension, *HEM/PRO* hematuria and/or proteinuria, *UTI/VUR* urinary tract infection/vesicoureteral reflux, *ABN* abnormal, *↓GFR* decreased glomerular filtration rate, *NC* nephrocalcinosis; GLOM/VAS, glomerular disease, vasculitis, *LABS* laboratory studies, *MICROALB* microalbuminuria; OTHER (as defined in Table 1)

*p < .001, †*p* < .05, ‡Early cohort as reference

Referrals for UTIs and VUR when considered together showed a significant decline in the latter cohort as compared to the earlier group (Tables 2 and 3). When considered separately, the change for each indication was similar. UTIs (alone) as the indication for referral decreased from 43 (8%) to 20 (4%) while VUR (alone) referrals decreased from 44 (9%) to 21 (4%) (*p* < .001 for both, data not shown).

**Table 3:** Referrals for Imaging

	Early cohort (2002–2004)	Late cohort (2011–2013)	
Referral Indication	n (% within cohort, % within category)	n (% within cohort, % within category)	χ [2], *p*; Odds Ratio [95% CI of OR]
Total	79 (15.5%)	96 (18.9%)	2.311, 0.128, 1.287 [0.929, 1.784]
ABN, US, OR CT IMAGING	20 (3.9, 25.0%)	31 (6.1, 32.0%)	2.543, 0.111, 1.592 [0.895, 2.832]
SK OR MCKD*	19 (3.7, 24%)	35 (6.9, 37.0%)	5.072, 0.024, 1.920 [1.079, 3.390]
HYDRO/DILATION UT	40 (7.8, 51.0%)	30 (5.9, 31.0%)	1.492, 0.222, 0.737 [0.452, 1.204]

*ABN* abnormal, *US* ultrasound, *CT* computed tomography, *SK* solitary kidney, *MCDK* multi-cystic dysplastic kidney, *HYDRO/DILATION UT*, hydronephrosis/dilation of urinary tract

*p < 0.05

Referrals for abnormal imaging findings did not show a significant change overall, though did increase from 15 to 19% of total referrals. The results from sub-group analysis of imaging referrals are shown in Table 3. Referrals for urinary tract dilation occurred less frequently in the later cohort as compared to the earlier group (not statistically significant), while referrals for solitary kidneys/multi-cystic dysplastic kidneys increased (*p* < 0.05). Miscellaneous imaging findings generated referrals more often in the recent time period but the difference did not reach significance.

## Discussion

There are limited data available on indications for nephrology referrals in the outpatient setting. Previous reports included inpatient as well as outpatient referrals, were limited to earlier decades (1977 to 2002) and categorized referrals based on the diagnosis rendered by the nephrologists rather than the referral indication given by primary care physician [10, 11]. Practice changes have occurred in the interim rendering the information less applicable to the current practice of pediatric nephrology. For example, one of these older analyses encompassed a time period before prenatal ultrasounds were performed or at least before they had become routine [10]. Additionally, with the inclusion of inpatient consultations in these older studies, it is not possible to determine what may have prompted primary care providers to request outpatient nephology consultations, the focus of the current study [10, 11]. There is a more recent study that addressed adherence to waiting time recommendations and reviewed outpatient referral to a single Canadian tertiary pediatric nephrology center during the period of 2007 to 2008 [4]. Their findings showed some similarities to ours with congenital abnormalities of the urinary tract and hydronephrosis (17%), hematuria or proteinuria (combined 22%), UTIs (12%) and elevated blood pressure (12%) accounting for almost 2/3 of the referrals.

Our study differs from these previous reports in that it offers a comparison of referral indications over a decade and focuses solely on the referring providers’ perception of the indication for pediatric nephrology input. Interestingly, we found a high rate of referral for abnormal imaging or laboratory results with a combined percentage of 18 and 25% for the early and late time periods, respectively. Comparison of our two cohorts demonstrated an increase in the number of referrals generated due to abnormal imaging in the latter group. These findings were not due to increasing recognition of antenatal hydronephrosis as the number of referrals for urinary tract dilation and hydronephrosis was reduced. Instead, referrals for solitary kidneys/multi-cystic dysplastic kidneys doubled. Referrals for various other abnormalities found on imaging such as isolated renal cysts, abnormal appearance or size of kidneys occurred at an increased frequency but due to our small numbers did not reach significance. Also as clinically suspected, referrals for abnormal laboratory studies occurred significantly more often in the recent cohort of patients. Primary care physicians faced with interpreting abnormal imaging or laboratory results may not feel qualified to advise parents on the significance of the findings. A recent survey of general pediatricians with 5 years or less of experience reported that 51% never or rarely cared for children needing nephrology services and 21% felt additional training in nephrology during residency would have been helpful [12]. Additionally, proximity to subspecialty care would be expected to factor into decision making. In the survey mentioned above, general pediatricians in rural settings were more comfortable managing subspecialty issues on their own as compared to those with local access to subspecialties [12]. In turn, parental expectations for access to pediatric subspecialties may be tempered in geographically remote areas [1].

Another novel finding demonstrated here was a reduction in the frequency of referral for UTIs and VUR. When considered together the proportion of referrals for these indications in our early cohort was 17% which was the same as the 16% reported by Radina et al., during the time period of 2007–2008 [4]. However, in our late cohort (2011–2013) the proportion of referrals for UTIs or VUR dropped in half for each category. In 2011 the American Academy of Pediatrics (AAP) issued guidelines which limited indications for imaging for VUR after UTIs with similar guidelines endorsed in the United Kingdom by the National Institute for Health and Clinical Excellence earlier in 2007 [8, 13]. Thus, our reduction in referrals for VUR may be due in part to a reduction in the frequency of imaging for VUR by community physicians. Additionally, the contribution of bowel and bladder dysfunction to the risk for recurrent UTIs has been increasingly appreciated and emphasized in educational forums for primary care physicians. Enhanced awareness of this connection may have empowered many to feel more comfortable addressing recurrent UTIs as well as various voiding issues on their own [8, 9, 14, 15]. Likewise, the reduction in referrals for urinary tract dilation and hydronephrosis might to some degree reflect increasing experience and evolution of practice in management of antenatal hydronephrosis [16, 17]. A recent multidisciplinary consensus statement on classification of antenatal and postnatal urinary tract dilation reflects a move toward standardization and should be helpful as it becomes more widely adopted [18].

Our study has several limitations. Instead of a full decade between time periods the gap between our study cohorts was approximately nine years. Electronic medical records were not available prior to July 2002 and review of paper records was not feasible due to offsite storage with high risk for incomplete data collection. However, the time span between the two cohorts was considered sufficient given evolving practices for issues such as prenatal hydronephrosis, UTIs and VUR between the study periods. Data were derived from a single general pediatric nephrology practice in a non-academic setting and may not reflect referral patterns in larger centers, or at centers with large catchment areas. However, the small number of nephrologists in our practice is not unusual for pediatric nephrology. The recent Pediatric Nephrology Workforce Survey reported an overall median group size of 4 with 46% of pediatric nephrologists practicing in groups of 3 or less [19]. Also, 27% reported practicing in a non-academic setting [19]. Additionally, use of the primary indication for referral likely resulted in some misclassification. The referral indication as specified by the primary care provider may not describe the actual finding(s) or diagnosis subsequently made by the nephrologist and secondary reasons for referral were not included. Our focus was the primary provider’s perception of the issue warranting a pediatric nephrology referral and not the final categorization by the nephrologist. It is possible that the observed decrease in referral for UTIs and VUR might be due to an increase in referrals to urology. Although this possibility cannot be excluded, the number of urology practices in our referral area did not change or enlarge between the two time periods. The reduction in referrals for voiding issues may reflect a change in our practice with more of these children being directed to urology rather than nephrology. Lastly, in rare instances children with known renal disease were referred to our practice after newly relocating to the area, or after pediatric nephrology services at a large nearby military base became unavailable. In such instances these children were considered new patients though in reality reflected a request for ongoing care.

The large proportion of referrals prompted by imaging and laboratory findings noted here supports the need for enhanced communication between providers. A survey of pediatricians conducted by the AAP in 2010 reported that 61% of rural and 46% of non-rural pediatricians perceived inadequate access to pediatric nephrologists [2]. Traditional access in the form of office consultation will continue to be a challenge as recent surveys by the AAP and the American Society of Pediatric Nephrology indicate that the work force is aging and limited in numbers and location [19]. Investigators from Texas and North Carolina have reported that phone consultations with pediatric subspecialists frequently allowed for continued local management and often obviated the need for an outpatient visit [20, 21]. These studies suggest that improved access to pediatric subspecialists electronically or via telephone could impact on decisions for referral and improve use of limited resources [20].

## Conclusion

Changes in indications for referral noted here likely reflect the evolution of clinical practice with regard to increased use of imaging, laboratory testing and updates in UTI/VUR practice guidelines over the two studied time periods of 2002–2004 and 2011–2013. These findings have implications for the education of primary care providers and pediatric nephrologists, particularly with regard to the use and interpretation of imaging studies. Whether increasing utilization of diagnostic studies is impacting referral patterns for other subspecialties is unknown. Further investigation along this line would help determine if our single center findings indicate changes in referrals to Pediatric Nephrology, or are reflective of broad changes in general pediatric practice. Given the ever increasing complexities of diagnostic modalities and the challenges inherent in providing subspecialty services, open communication between primary care providers and pediatric nephrologists is critical to the provision of necessary and timely services.

## Abbreviations

OR: odds ratio
UTI: urinary tract infection
VUR: vesicoureteral reflux
